# Supplementary material for: Improving genetic risk prediction across diverse population by disentangling ancestry representations
Source: Commun Biol. 2023 Sep 22;6:964. doi: 10.1038/s42003-023-05352-6 (PMC10517023; doi:10.1038/s42003-023-05352-6)
Supplement: Supplementary file 2 — Supplementary Information [file 42003_2023_5352_MOESM2_ESM.pdf]

Improving genetic risk prediction across diverse population by disentangling ancestry representations: Supplementary Information

Supplementary Figure 1. Neural network architectures. The top row represents architecture for Disentangling autoencoder (**DisentglAE**), the middle row represents architecture for supervised Neural Network (**NN**), and the bottom row represents Adversarial learning (**Adv**). For all the networks except Adv Critic (bottom row, right), the genotype data is presented to the network as the first input data with dimension (input dim) of 3892 for the ADSP and 4967 for the UKB. For Adv Critic, the input dimension is the output of the FC_2 layer of Adv Backbone. Linear Layer represents multi-layer perceptron, and ReLU represents Rectified Linear Unit, a nonlinear activation function. For DisentglAE, $\mathbf{z_d}$ dim and $\mathbf{z_a}$ dim represents latent dimensions respectively for phenotype-specific representation and ancestry-specific representation.

Layer	DisentglAE
FC_1	Linear Layer (input dim, 400), ReLU
FC_2	Linear Layer (400, 200), ReLU
FC_{31} ,FC_{32}	Linear Layer(200, z_d dim), Linear Layer(200, z_a dim)
FC_4	Linear Layer (z_d + z_a dim, 200), ReLU
FC_5	Linear Layer (200, 400), ReLU
FC_6	Linear Layer (200, input dim), ReLU

Layer	NN
FC_1	Linear Layer (input dim, 200), ReLU
FC_2	Linear Layer (200, 100), ReLU
FC_3	Linear Layer(100, 20), ReLU, dropout (p = 0.5)
FC_4	Linear Layer(20, 20), ReLU, dropout (p = 0.5)
FC_6	Linear Layer (20, 1)

Layer	Adv Backbone
FC_1	Linear Layer (input dim, 200), ReLU
FC_2	Linear Layer (200, 20), ReLU
FC_3	Linear Layer (20, 1)

Layer	Adv Critic
FC_1	Linear Layer (20, 10), ReLU
FC_2	Linear Layer (20, 1)

Supplementary Note 1

In **Supplementary Figure 2**, we present the extended results that include a comparison of the proposed DisPred with Disengl-AE, Adv (adversarial learning to capture domain invariant representations where we treated ancestry as domains) and the models trained using African American population (AFR). We present the result for the ADSP cohort, where we consider four test datasets: i) all test participants, ii) admixed participants, iii) European participants, and iv) non-European participants, primarily AFR for the ADSP. For all cases except for the European participants, the proposed Dispred leads to the best result. When the analysis is restricted to European participants, as shown in panel C, PRS-Lasso EUR achieves the best outcome, and the proposed method produces the second-best result for both datasets. In all cases except the proposed method, all the neural network-based methods, including Adv, demonstrate poor generalizability. In **Supplementary Figure 3**, we present the results for UKB EUR (n =423,357) obtained from DisPred, compared to other models trained on ADSP EUR. This is to evaluate different models'

performance on dataset shift (or distribution shift), i.e., the application of models trained on one cohort to participants of a separate cohort. Across all cases, DisPred achieves the best result. In **Supplementary Table 1**, we present the robustness analysis, using the different random seeds to split our dataset and conduct the experiments. In this analysis, seed 1 represents the results presented and discussed earlier, and seed 2 represents a different data split. As shown in the table, our presented method DisPred achieves superior performance for both seeds.

Supplementary Figure 2. Prediction accuracy (AUC) for different models trained on European participants (PRS-Clumping EUR, PRS-Bayes EUR, PRS-Lasso EUR, NN EUR), trained on African participants (PRS-Lasso AFR, NN-AFR), Adversarial Neural Network trained on all data (Adv), Disentangling Autoencoder (Disentgl-AE), and DisPred trained on all data) on different subsets of the test dataset: all participants (A), admixed participants (B), EUR participants (C), and participants from non-European ancestries (D) in the test dataset for the ADSP cohort.

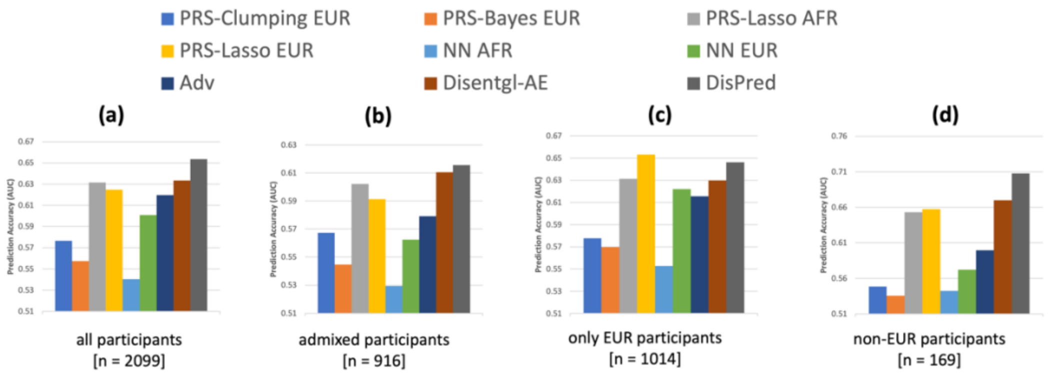

Supplementary Figure 3. Prediction accuracy (AUC) for different models trained on European participants (PRS-Clumping, PRS-Lasso, Neural Network (NN)) and proposed DisPred trained on all data on European participants (UKB EUR) from the UKB cohort.

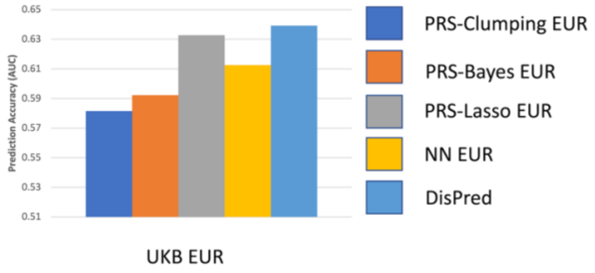

Supplementary Table 1. Prediction accuracy (AUC) for different models (PRS-Clumping EUR, PRS-Bayes EUR, PRS-Lasso EUR, NN EUR), and DisPred trained on all data) on two different subsets of the test dataset: African participants (ADSP AFR), and admixed individuals, for two distinct dataset splits.

Test subsets	Random seed	PRS-Clumping EUR	PRS-Bayes EUR	PRS-Lass EUR	NN EUR	DisPred
ADSP AFR	Seed 1	0.5385	0.5255	0.6575	0.5722	0.7078

	Seed 2	0.5392	0.5314	0.6643	0.5812	0.6923
ADSP admixed	Seed 1	0.5673	0.5446	0.5914	0.5625	0.6169
	Seed 2	0.5312	0.5387	0.5645	0.5604	0.6012
