# Supplementary material for: Improving genetic risk prediction across diverse population by disentangling ancestry representations
Source: Commun Biol. 2023 Sep 22;6:964. doi: 10.1038/s42003-023-05352-6 (PMC10517023; doi:10.1038/s42003-023-05352-6)
Supplement: Supplementary file 3 — Description of Additional Supplementary Files [file 42003_2023_5352_MOESM3_ESM.pdf]

Description of Additional Supplementary Files

File name: Supplementary Data 1
Description: Source data behind the graphs in Figure 3.

File name: Supplementary Data 2
Description: Source data behind the graphs in Figure 4.

File name: Supplementary Data 3
Description: Source data behind the graphs in Figure 5.

File name: Supplementary Data 4
Description: Source data behind the graphs in Supplementary Figures 1 and 2.
